# Macrophage Subsets in Obesity, Aligning the Liver and Adipose Tissue

**DOI:** 10.3389/fendo.2020.00259

**Published:** 2020-04-29

**Authors:** Anneleen Remmerie, Liesbet Martens, Charlotte L. Scott

**Affiliations:** ^1^Laboratory of Myeloid Cell Heterogeneity and Function, VIB-UGent Center for Inflammation Research, Ghent, Belgium; ^2^Department of Biomedical Molecular Biology, Faculty of Science, Ghent University, Ghent, Belgium; ^3^Laboratory of Myeloid Cell Ontogeny and Functional Specialization, VIB-UGent Center for Inflammation Research, Ghent, Belgium

**Keywords:** macrophages, adipose tissue, non-alcoholic fatty liver disease (NAFLD), single-cell, heterogeneity, liver, NASH (non-alcoholic steatohepatitis)

## Abstract

The increasing prevalence of obesity is accompanied by a rising incidence in metabolic syndrome and related pathologies such as non-alcoholic fatty liver disease. Macrophages are hypothesized to play central roles in these diseases, through their role as inflammatory mediators and as such are thought to be potential targets for future therapies. Recently, single cell technologies have revealed significant heterogeneity within the macrophage pool in both liver and adipose tissue in obesity. Thus current efforts are focused on dissecting this heterogeneity and understanding the distinct functions of the individual subsets. In this review, we discuss the current knowledge regarding macrophage heterogeneity, ontogeny and functions in the context of obese adipose tissue and fatty liver disease and attempt to align the distinct populations described to date.

## Introduction

The transition to a more sedentary lifestyle coupled with a higher caloric intake has led to an enormous rise in incidences of obesity over the past decades. Simultaneously, this has led to a dramatic increase in the number of patients suffering from numerous obesity-linked metabolic disorders including insulin resistance and type 2 diabetes, cardiovascular disease and non-alcoholic fatty liver disease (NAFLD). In fact, since 1975, obesity levels have almost tripled worldwide and according to the World Health Organization (WHO) this accounted for ~8% of deaths in 2017 largely due to cardiovascular disease. Chronic low-grade inflammation has been suggested to be related to many of the co-morbidities of obesity including type 2 diabetes, NAFLD, steatohepatitis and cardiovascular disease [reviewed in (1)], leading to the concept of “metainflammation” (2). This is characterized by abnormal cytokine production, activation of inflammatory signaling pathways and increased acute phase reactants (3). Macrophages, as cells of the innate immune system, are thought to contribute significantly to metainflammation across obese tissues [reviewed in (4)]. Here, macrophages have been suggested to alter their phenotype toward a more pro-inflammatory profile and this has been proposed to be detrimental in the long-term, driving fibrosis and tissue damage (5, 6). More recently, the idea that these macrophages may be generated as (initially) a tissue protective mechanism, to deal with the increased lipid load has also been proposed (7). In recent years, it has become evident that there are multiple subsets of macrophages present in both obese adipose tissue and the fatty liver that may contribute differently to the pathogenesis of disease. What remains unclear is whether these represent an altered phenotype of tissue-resident macrophages (Res-macs) or newly recruited populations of macrophages. In the latter case how these recruited macrophages relate to their tissue-resident counterparts also remains unclear. Answering these questions will be crucial to deciphering the different functions of the macrophages and understanding how best to target them therapeutically. While our understanding of macrophage heterogeneity, has been greatly improved through the use of single cell technologies including single cell RNA sequencing (scRNA-seq), there is often little consensus between studies with authors often using expression of different genes to identify subsets and giving the subsets identified distinct names. In this review, we will discuss what is currently known regarding the macrophage subsets present in the liver in non-alcoholic fatty liver disease and in obese adipose tissue, their origins and their specific functions. In addition, where possible, we attempt to align the different populations identified to date. For the most part the studies discussed here have been conducted in murine models unless stated otherwise (Table S1).

## Tissue-Resident Macrophages

Under homeostatic conditions, tissue-resident macrophages (Res-macs) represent the majority of macrophages in the body. These are generated alongside their tissue of residence, typically during embryogenesis and as such derive, at least initially, from embryonic progenitors including yolk-sac macrophages and fetal liver monocytes [reviewed extensively in (8–10)]. As the tissues grows in the 1st weeks of life, bone-marrow derived monocytes can also engraft in some tissues and generate Res-macs (8, 11). In most tissues, once organ growth has ceased, the Res-macs are maintained independently from any significant input from circulating progenitors, but rather through local proliferation of existing macrophages, although there are a few exceptions to this including the gut, dermis, heart and lung interstitial macrophages which are continuously replaced from the BM throughout life [reviewed in (8, 9, 12)]. Additionally, in old-age, BM monocytes may also start to contribute again to the Res-mac pools in some tissues, with a slight increase in BM-derived macrophages being observed in the spleen and peritoneal cavity between 36 and 46 weeks after tamoxifen labeling (13). Notably, in the lung and liver, the specific origin of the Res-macs, termed alveolar macs (Res-AMs) and Kupffer cells (Res-KCs) respectively, whether yolk-sac macrophages, fetal liver monocytes or bone marrow monocytes, does not appear to significantly affect the transcriptional profile of these macrophages or their ability to self-renew provided they are generated under homeostatic conditions (11, 14). This conditioning of progenitors enabling the development of Res-macs under homeostatic conditions, is proposed to result from the interactions between the progenitors/macrophages and the specific cells in their local environment or “niche” including stromal cells, endothelial cells and structural cells (8, 15) and indeed crosstalk between macrophages and fibroblasts has already been demonstrated *in vitro* under both healthy and fibrotic conditions (16, 17), while *in vivo*, many of the signals driving monocyte recruitment and differentiation into Kupffer cells following depletion have already been elucidated including Il1β, TNFα, DLL4, NOTCH, LXRα, and BMPs (18, 19).

Regarding their functions, Res-macs fulfill central roles in tissue homeostasis and immunity through their constant surveillance and their ability to clear foreign substances, dead cells and debris. This clearance is mediated through recognition of pathogen-associated molecular patterns (PAMPs) or damage-associated molecular patterns (DAMPs) by pattern recognition receptors (PRRs) present on their cell surface. In addition to physically clearing the tissue of pathogens, dead/dying cells and other debris, Res-macs can also express multiple cytokines which contribute to the activation of other innate immune cells and the adaptive immune system. Notably, through their unique transcriptomic profiles imprinted by signals from the local niche (8, 15), Res-macs are also proposed to play additional roles in maintaining tissue homeostasis, performing several “accessory” functions depending on the needs of their tissue of residence (20). These tissue-specific transcriptional profiles have been shown to be driven through expression of “master” transcription factors, which are imprinted in the macrophages by signals from their local niche (21, 22). For example, the transcriptional profiles of Res-KCs in the liver and Res-AMs in the alveolar space of the lung are both enriched for genes associated with a lipid metabolism function (21, 23), with the lipid metabolism profile of KCs being driven at least in part through expression of LXRα (23), which is imprinted on KCs by liver sinusoidal endothelial cells and hepatic stellate cells (18, 19). Similarly, Res-macs in the liver and spleen are enriched for genes necessary for iron processing, a function regulated by the master transcription factor SpiC (23–25).

With the identification of the substantial role played by the niche in regulating Res-mac gene expression and hence functionality, one of the key questions in the macrophage field today is how does the niche change in inflammation and how does this impact Res-mac survival and function? Notably, a population of microglia with a distinct transcriptional and functional profile have recently been described in aged mice (20 months). These microglia contain lipid droplets and were hence termed lipid droplet-containing aged microglia (LDAMs) (26). As there is limited input of HSCs to the microglia pool 46 weeks after tamoxifen administration (13) these LDAMs are suggested to be a population of Res-microglia, suggesting that Res-macs may indeed respond to changes in their local environment as occurs in aging or inflammation. However, as the origin of LDAMs was not formally tested in this study, it remains to be seen if these are indeed altered Res-macs or a newly recruited population (26). Nevertheless, understanding how the niche changes and how this may alter the macrophages is relevant question especially in the context of obesity and metabolic disease, where, for example, changes in hepatic stellate cells and myofibroblasts in the liver have been already been reported in NASH and fibrosis in both mouse and human samples (27, 28).

## Recruited Macrophages

Alongside Res-macs, when homeostasis is perturbed, for example due to inflammation and/or infection, bone marrow monocytes are recruited to the tissue, where they subsequently differentiate into macrophages. In some instances these cells represent a short-lived transient population that are recruited during the inflammation/infection but are typically lost a few days after return to homeostasis (29–32), thus from herein these will be termed “temporary macrophages” (Temp-macs). However, bone marrow monocytes can also engraft and become long-lived resident macs under non-homeostatic conditions (32–36). When this happens in the brain due to genetic models or irradiation substantial differences are observed between the profiles of these recruited Res-macs compared with the embryonically-derived Res-microglia, including expression of the microglia-identity gene *Sall1* (33–35). Thus, we propose to call these Res-macs recruited under non-homeostatic conditions inflammatory Res-macs (infRes-macs). Whether these differences in brain microglia are due to timing and hence a potentially altered niche (for example adulthood vs. embryo) or due to intrinsic differences in the progenitors remains to be investigated. Notably, BM-derived KCs engrafting after genotoxic injury also display some (albeit minor) differences in their transcriptional programs compared with those engrafting under homeostatic conditions following DT mediated depletion (11, 37), suggesting the altered niche may be the most important factor at play. In the lung in the context of influenza infection, Res-AMs were found to be largely unchanged by the virus but reduced in numbers, which led to the development of BM-derived infRes-AMs during the infection with distinct functions, transcription and epigenetic profiles as compared with the Res-AMs (36). Notably, with time spent in the lung niche following recovery from infection, these differences were gradually lost and thus the infRes-AMs more closely resembled the Res-AMs with time (36). In the liver in a model of NASH, we also observed BM-monocytes differentiating into infRes-KCs that were maintained for at least 4 weeks after return to homeostasis (32). However, how similar these infRes-KCs were to the Res-KCs was not investigated (32) and remains an open question. Given the observation that infRes-macs can have significantly altered profiles and hence altered functionality compared with Res-macs, these macs should be discriminated from one another. This could be especially important in disease settings where one might aim to promote Res-macs over newly recruited infRes-macs or *vice versa* depending on their specific functions.

The recruitment of both Temp-macs and infRes-macs to supplement the original Res-macs in inflammation raises multiple questions. For example, why do we recruit macrophages in non-homeostatic conditions? Does this mean that Res-macs are not plastic enough to be able to deal with the insult? Do Res-macs alter their profiles as has been previously suggested or are changes within the total macrophage pool due solely to the recruitment of Temp-macs and infRes-macs? Indeed the influenza study would suggest the original Res-macs are not substantially altered by the virus but rather cannot maintain their numbers and hence require input from the BM (36). Is this true in obese tissues? Are infRes-macs required to maintain macrophage numbers as some Res-macs are lost and proliferation rates are not sufficient to replenish the pool? If so, how similar are these infRes-macs to their original Res-mac counterparts? Moreover, how distinct are infRes-macs and Temp-macs? Do these represent distinct populations of macrophages entering the tissue or can some Temp-macs also become infRes-macs? In the context of obesity, it will be important to address these questions to understand (i) which subsets are important in the pathology, (ii) which signals drive the recruitment and differentiation of the different subsets, and (iii) how we could target the required subsets therapeutically. Crucially, it will be important to understand which subsets are found across multiple labs, models, and indeed species to be able to understand which populations could be clinically relevant. With this in mind, below we will discuss the current knowledge regarding the distribution and origins of different macrophages subsets and their functions in obese tissues and attempt to align the subsets identified by different groups. However, as will become clear, this is not always straight-forward and as a result there are still many unanswered questions regarding these cells in obesity.

## Macrophage Subsets in Nafld and NASH

NAFLD is the hepatic manifestation of metabolic syndrome. It currently represents the most common etiology of chronic liver inflammation in the western world. Due to the lack of treatment options available, it is predicted to be the primary reason for liver transplantation in the western world by 2030 (38–41). The current lack of therapies for patients with NAFLD stems from the fact that NAFLD is a complex disease, consisting of different stages ranging from simple steatosis (retention of fat in the liver) to non-alcoholic steatohepatitis (NASH), cirrhosis and even hepatocellular carcinoma (HCC). While patients with simple steatosis are largely asymptomatic, the progression to NASH, fibrosis and eventual cirrhosis leads to the need for liver transplantation. Notably, this progression from NAFLD to NASH does not occur in all patients and the mechanisms underlying this transition remain largely unknown. It has been proposed that multiple “hits” coming from the gut and adipose tissue may account for this progression (42), with one of the hits being the ensuing inflammation potentially driven by macrophages in response to the increased lipid load and hepatocellular death.

During steady state, the predominant macrophage population present in the liver is the resident Kupffer Cells (Res-KCs). They are one of the largest populations of Res-macs present in the body and reside with part of their body in the liver sinusoids where they are in close contact with liver sinusoidal endothelial cells (LSECs) and part in the liver parenchyma where they are in close contact with hepatic stellate cells (HSCs) and hepatocytes (19). In mice, KCs can be identified by their specific expression of the C-type lectin, CLEC4F (11). In addition, once resident in the liver, KCs also express TIM4 and thus the combination of CLEC4F and TIM4 is very useful to discriminate between Res-KCs (CLEC4F^+^TIM4^+^) and recently recruited KCs, on their way to becoming resident (CLEC4F^+^TIM4^−^) and Temp-macs (CLEC4F^−^TIM4^−^) that can either differentiate into KCs or be lost from the tissue upon return to tissue homeostasis (11, 32). In humans, the best markers of Res-KCs compared with the other mac populations present in the liver are still being elucidated, although *CD163, TIMD4*, and *MARCO* expression have been suggested to identify Res-KCs (28, 43).

**Figure 1:**
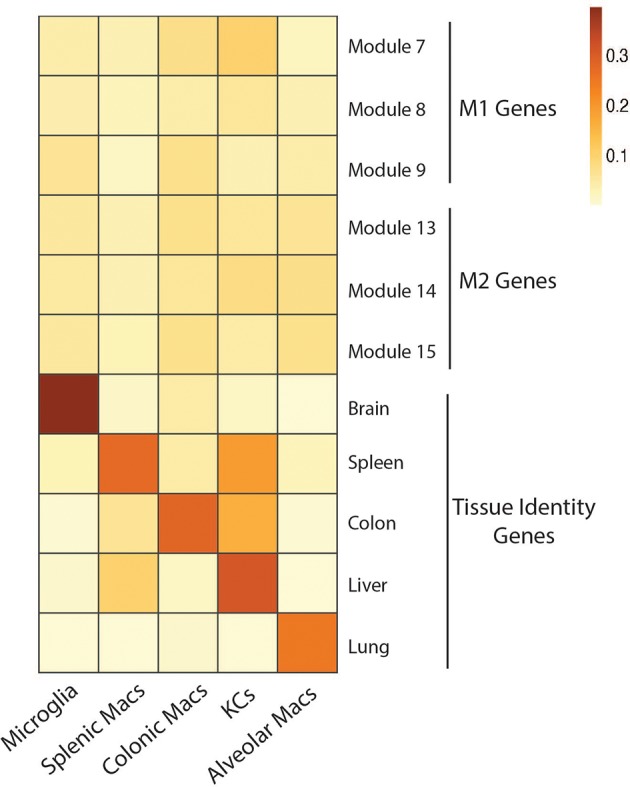
No overt M1 or M2 activation profile in tissue resident macrophages in homeostatic conditions. Expression of gene modules associated with M1 (module 7, 8, 9) and M2 (module 13, 14, 15) human macrophages (55) by microglia, splenic macrophages, colonic macrophages, Kupffer cells and Alveolar macrophages isolated from mice under homeostatic conditions (23). Expression of core identity genes of each tissue resident macrophage population (23) were also visualized as a control (Genes associated with each module and population can be found in Table S2). To generate the heatmap, mouse gene symbols were used as input for the AUCell R package. In brief, AUCell allows you to identify cells with active gene sets (e.g., signatures, gene modules) in single cell RNA-seq data by first ranking the genes from highest to lowest value per cell. Next, area under the curve (AUC) is calculated to determine whether the gene set is enriched at the top of the gene-ranking for each cell. The output is a matrix with an AUC score for each gene set in each cell. For the heatmap we calculated the mean AUC of all cells.

In the context of NAFLD, Res-KCs have been proposed to drive the progression from NAFLD to NASH through their role as inflammatory mediators [recently reviewed in (6)]. This hypothesis stems from studies suggesting the balance between M2- and M1-like hepatic macrophages is altered in NAFLD and that promoting more M2-like macrophages may be beneficial in NAFLD (44–48). In addition, studies depleting macrophages using models including LysM-driven genetic models and clodronate liposomes have further fueled the hypothesis that Res-KCs drive the development of hepatic steatosis, insulin resistance, liver damage and inflammation (49–53). However, two main findings are now altering this line of thinking. Firstly, it is now clear that the M1/M2 polarization states are not sufficient to describe the complex milieu of signals which imprint macrophage phenotypes and functions *in vivo* (54). Indeed, under homeostatic conditions, the transcriptional profile of Res-KCs and other tissue Res-macs do not resemble those of either M1 or M2 macrophages (Figure 1, Table S2). Moreover, it has become clear that Res-KCs are not the only population of macrophages in the fatty liver, with a number of Temp-macs being recruited from the bone marrow in a CCR2-dependent manner that are also thought to play a role in the progression to steatohepatitis and fibrosis (56–58). Thus, it is not yet clear if Temp-macs or Res-KCs are contributing to the phenotypes observed when total macrophages are manipulated using such non-KC-specific models as described above and hence the exact roles played by Res-KCs in NAFLD/NASH remain to be understood. In addition, in mice fed a methionine-choline deficient (MCD) diet to induce NASH, we demonstrated that CLEC4F^−^ Temp-macs are recruited to the liver and that some of these give rise to CLEC4F^+^TIM4^−^ infRes-macs (32). Thus, potentially, there could be at least 3 distinct subsets of macs with different transcriptional profiles and functions in NAFLD/NASH. However, as mice fed the MCD diet develop NASH symptoms but do not gain weight (32), whether these infRes-Macs are also found in more biologically relevant models of NAFLD/NASH or indeed in human NAFLD/NASH remains to be seen as discussed below.

With these caveats of earlier studies in mind, what have we learnt in recent years regarding the subsets and roles of liver macrophages in NAFLD/NASH? Intriguingly and contrary to the widely accepted hypothesis, a recent study has found that neither obese human macrophages nor high-fat diet fed (12 weeks) murine liver macrophages (purified as adherent cells following tissue digestion, percoll gradient and plating) altered their expression of a standard panel of activation associated genes including *Tnfa, Il1b, Il6, Il10, Tgfb1, Ccl2, Ccl5, Itgax, Cd80, Socs3, Chil3, Arg1*, or *Egr2* (59). This therefore raises the question whether Res-KCs do respond to the fatty liver environment and mediate the inflammation as originally proposed or if perhaps they behave more like the Res-AMs in the lung during influenza infection (36). However, as here there was no discrimination between Res-KCs and other liver macs (59) it is difficult to draw any conclusions regarding the roles of these cells, perhaps Res-KCs are not activated or perhaps they are but their numbers are reduced in NAFLD leading to the majority of total macs being Temp-macs without an overtly activated profile. Thus, further work is required to answer these questions.

The recent advances in scRNA-seq studies have started to shed light on the heterogeneity of macrophages present in the NAFLD/NASH liver. One recent study employing scRNA-seq of CD45^+^Ly6G^−^ myeloid cells isolated from mice fed a western diet supplemented with fat, sugar and cholesterol for 16 weeks identified 3 distinct clusters of recruited macrophages (termed Mo-MFs) and a cluster of likely Res-KCs (57). All of these macrophage populations, as well as cDC2s and bone marrow monocytes in these mice expressed lower levels of *S100a8* and *S100a9* encoding the inflammatory protein Calprotectin compared with the same populations in control mice fed a normal diet, suggesting they may have anti-inflammatory properties (57). Fitting with this, challenge of these mice with an overdose of paracetamol as a model of acute liver injury resulted in attenuated disease compared with normal diet fed controls (57). While a similar response of recruited macrophages and Res-KCs as observed here (57), would be consistent with the relative unresponsiveness of total liver macrophages observed by the Aouadi lab (59), there are a few remaining questions surrounding this study. The presence of recruited macrophage populations in the control mice (albeit at lower frequencies than the NAFLD mice) is rather unexpected especially as some of these populations seem to be more abundant than the KCs (57). This raises the question if the control mice are completely healthy? If so, the question becomes what are these recruited macrophage populations in the control mice and where are they located? In addition, a key question is whether the distinct clusters of recruited macrophages identified truly represent distinct subsets of macrophages or rather one population existing along a gradient of activation and/or developmental stages? Regarding the relative paucity of Res-KCs, while the authors attribute this to digestion and cell isolation, it raises the question as to whether the Res-KCs isolated are representative of the total Res-KC pool in NAFLD/NASH? Moreover, as the origins of these cells were not studied it remains to be seen if these are truly Res-KCs or if any infRes-KCs may also be found in the NAFLD/NASH liver.

A second recent study also employing scRNA-seq but this time using mice fed an amylin (ALMN) diet for 20 weeks to induce NASH identified distinct clusters of macrophages in the NASH liver (27). Here one population of recruited macrophages alongside two KC subsets were identified. The two KC subsets were identified as KCs based on expression of *Cd5l* and the two subsets were distinguished from each other based on expression of *Trem2, Cd9*, and *Gpnmb* (27). The KC population expressing all of these genes was only present in the NASH condition and was therefore termed NASH-associated macrophages or NAMs (27). The expression profile of these NAMs was significantly different from healthy Res-KCs (27), but the healthy Res-KC profile was largely maintained in the non-NAM KCs in the ALMN diet (27), further supporting the idea that at least some of the Res-KCs may not be altered in NAFLD/NASH. As the relationship between the two KC subsets identified in the ALMN-fed mice was not studied (27), it is not clear if the NAMs represent a recruited population of infRes-KCs, while the non-NAM KCs represent Res-KCs that do not alter their phenotype in NASH or if the NAMs are a population of Res-KCs that have altered their transcriptome due to the NASH environment. This will of course be important to address as targeting such macrophages either to assess their functions or later on in the clinic would require different approaches in each case. Notably while further study is required to understand the nature of these macrophage subsets, macrophages with a phenotype similar to the NAMs have also recently been identified in fibrotic human livers (28) indicating the potential relevance of these macs in human disease although not necessarily restricted to NASH as the name NAMs would suggest. The macrophages identified in fibrotic human liver tissue were rather termed scar-associated macrophages (SAMs) and were specifically located around the fibrotic scars (28). Interestingly the SAMs were distinct from KCs further highlighting the need to investigate the designation of NAMs as KCs in the mouse (27).

**Figure 2:**
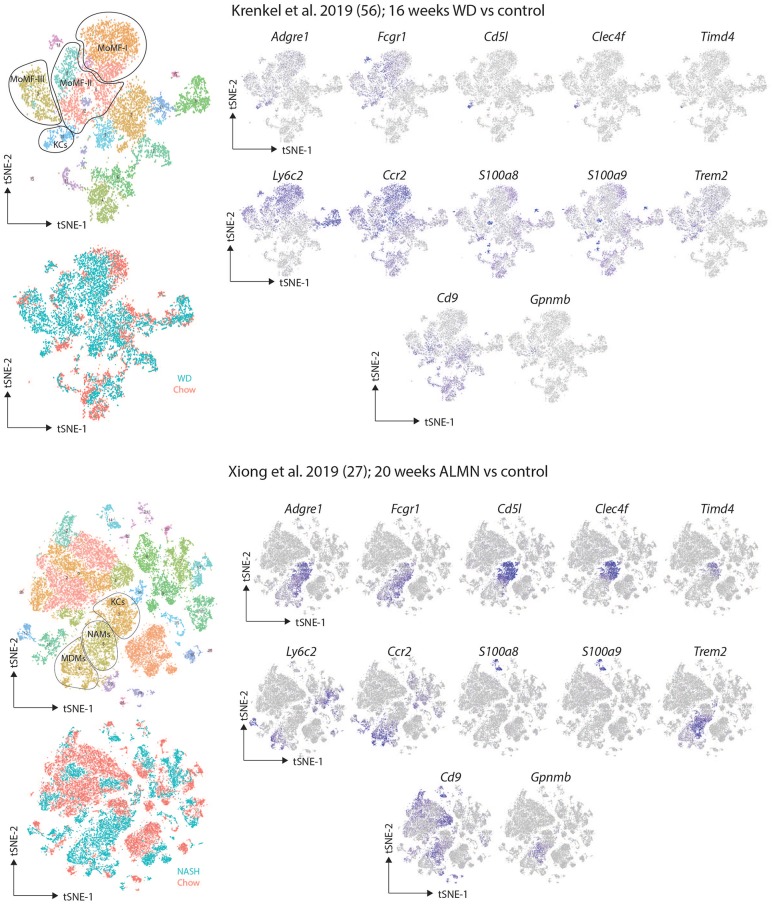
Comparison of murine scRNA-seq datasets on macrophages in NAFLD/NASH. Publicly available scRNA-seq datasets from (27, 57) were downloaded and tSNE maps recreated as close to the original as possible with the information provided. The distinct clusters of macrophages and KCs as identified in the original studies (27, 57) were identified and expression of the indicated markers assessed.

Despite the advances in understanding macrophage heterogeneity, an unfortunate feature of these studies is that it is not always easy to link the subsets identified in the different studies. This is further complicated by each study giving a different name to the macrophage populations they identify. A crucial next step will therefore be to understand what subsets and profiles are shared across NAFLD/NASH models and between species and how best to identify them for downstream functional studies which are still lacking. To try to align these studies, we have downloaded the data from the two reports and recreated the tSNE plots (27, 57). While the tSNE plots are not exact replicates, the same clusters identified by the two groups could be identified in our recreations based upon the data provided and the gene lists published for each population (Figure 2). Expression of a set of markers proposed as Res-KC markers (*Clec4f, Timd4, Cd5l*), general macrophage markers (*Adgre1, Fcgr1*), monocyte markers (*Ccr2 and Ly6c2*) and the markers proposed by the studies (*S100a8, S100a9, Cd9, Trem2*, and *Gpnmb*) were assessed (Figure 2). Interestingly, the subsets identified in both studies as recruited macrophages expressed relatively low levels of the macrophage markers *Adgre1* (encoding F4/80) and *Fcgr1* (encoding CD64) while still expressing the monocyte genes *Ly6c2* (encoding Ly6C) and *Ccr2*, thus perhaps some of these populations may represent monocytes or monocytes transitioning to macrophages rather than fully differentiated macrophages (Figure 2). This would also explain their presence in the healthy controls in the study from the Tacke lab (57). In terms of expression of the KC-specific markers, it appears that some of the NAMs do not express these genes suggesting they may indeed represent Temp-macs (Figure 2). While further studies will be required to confirm this, this could indeed suggest that Res-KCs are not significantly altered in NAFLD/NASH but rather that recruited macs in NASH have a distinct phenotype from Res-KCs. Regarding the presence of infRes-macs, in both studies a population of KCs exist that do not express *Timd4* (encoding TIM4), a marker of long-term residence of KCs in the liver (Figure 2). This could suggest that like in the MCD-induced NASH model, infRes-KCs may be generated with an altered profile to Res-KCs. However, as coverage of *Timd4* expression is not always 100% even in homeostatic conditions (23) and we do not yet fully understand the signals driving *Timd4* expression in KCs, this will need to be confirmed with fate-mapping studies. While *S100a8* and *S100a9* expression was not seen in the study from Xiong and colleagues (Figure 2), what is notable is that the *Trem2, Cd9, Gpnmb* signature of NAMs (27), that is also found in macrophages from human fibrotic tissue (28) can also be found in some cells in one of the recruited macrophage clusters (mo-mf-II) in the study from Krenkel et al., notably, in the cells expressing lower levels of *Ly6c2* and higher levels of *Adgre1* and *Fcgr1* compared with the other populations designated as recruited macs (Figure 2), suggesting that this may be a truly conserved signature of Temp-macs in fibrosis. Thus, understanding the functions of these cells represents an important question for future studies.

Another key question is if Res-KCs are not functioning as inflammatory mediators in NAFLD as these recent studies may suggest, do they play any role? One possibility is that Res-KCs may function to metabolize the ectopic lipid in NAFLD, as under homeostatic conditions Res-KCs have been shown to express a module of genes associated with lipid/cholesterol metabolism (11), although this remains to be tested directly. In line with other roles for Res-KCs, Morgantini et al. proposed that liver macrophages would function in NAFLD through their production of non-inflammatory factors including *Igfbp7* regulating liver metabolism (59). While an interesting concept that should be followed up, it will also be important to determine which of the liver macrophage subsets in NAFLD express *Igfbp7 (*Res-KCs or recruited macs) and how specific this expression is across the liver. For example, do any of the niche cells of the liver such as HSCs and LSECs which express higher levels of *Igfbp7* than Res-KCs under homeostatic conditions (unpublished data) also express *Igfbp7* in NAFLD/NASH and what role does this play?

**Figure 3:**
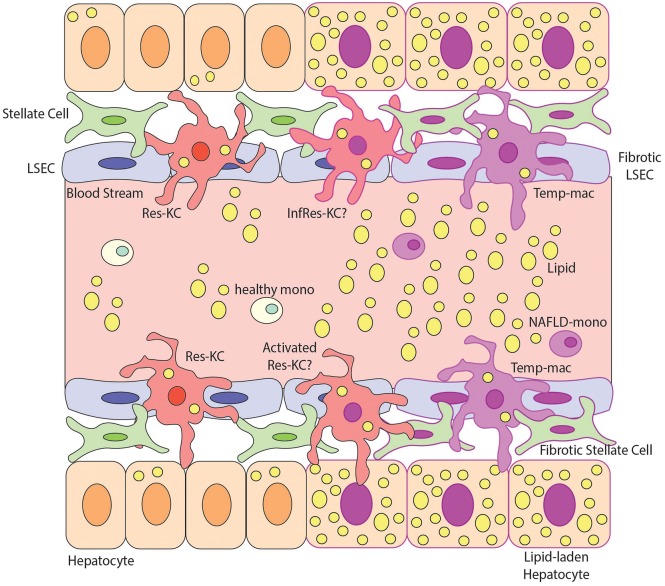
Macrophage Heterogeneity in NAFLD and NASH. This figure demonstrates possible macrophage heterogeneity present during NAFLD/NASH. During steady state the main macrophage population present in the liver is resident Kupffer Cells (Res-KCs) which are in close contact with the liver endothelial cells (LSECs), stellate cells and hepatocytes. Healthy monocytes pass through the blood flow of the liver sinusoids daily and typically do not engraft unless the niche becomes available. During NAFLD there is an increase in lipids in the liver and subsequently hepatocytes become lipid laden. The lipemic environment also contributes to stellate cell and endothelial cell activation likely altering the macrophage niche. Multiple populations of macrophages have been identified in the NAFLD liver but the relationship between them remains unclear. For example, does the lipemic environment activate the Res-KCs (activated Res-KCs) or do monocytes (which have been shown to have an altered transcriptional profile) recruited to the liver in NAFLD engraft and give rise to infRes-KCs, with a similar profile to Res-KCs but showing signs of the altered environment. Additionally, in NAFLD, monocytes have been shown to engraft and generate Temp-macs, although how distinct these are from either Res-KCs, activated Res-KCs or InfRes-KCs remains to be studied. For example, an interesting population of macrophages expressing *Cd9, Trem2*, and *Gpnmb* in murine NASH and human fibrotic livers have been described (27, 28) but whether these represent activated Res-KCs, infRes-KCs or Temp-macs remains to be formally examined.

Taken together, this significant level of heterogeneity within the macrophage pool in NAFLD/NASH (Figure 3) highlights the need to understand the specific functions of the different macrophage populations identified. In addition, with the possibility that some monocytes infiltrating the liver in NAFLD/NASH may give rise to infRes-KCs (Figure 3), this brings into question the strategy to target all recruited cells in NAFLD through CCL2 inhibitors (60), as potentially infRes-KCs and Temp-macs may play distinct roles in disease pathology. Thus, further investigation into the biology of this heterogeneity is warranted, especially in terms of understanding the functional contribution of the conserved subset of putative Temp-macs expressing *Cd9, Trem2*, and *Gpnmb* found across multiple models and in human fibrotic tissue as well as the location of these subsets in the tissue and their interactions with the local niche. Of note, one study in *Trem2* KO mice fed a western diet found reduced pathology in the KOs compared with WT controls (61). While this was attributed to macrophages in the adipose tissue (61) (see below) it is also possible that this phenotype was due to the lack of *Trem2*-expressing macrophages in the fatty liver, further highlighting the need for functional studies with these populations.

## Macrophage Subsets in Obese Adipose Tissue

The adipose tissue consists mainly of adipocytes and functions as an energy store but also produces hormones, for example those regulating lipid metabolism and hunger satiety. Adipose tissue macrophages (ATMs) are spread throughout the adipose tissue with a significant proportion in lean mice being found associated with the vasculature and hence recently termed vasculature-associated macrophages or VAMs (62). VAMs are identified on the basis of their expression of CD206 and CD11b and can be subdivided into two subpopulations (VAM1 and VAM2) based on MHCII and TIM4 expression, with VAM2s expressing higher levels of TIM4 (62). In addition a population of CD206^int^CD11b^+^CD64^+^CD11c^−^ pre-VAMs and CD206^int^CD11b^+^CD11c^+^CD64^+^ double positive (DP) macrophages were also identified in this study although their localization was not investigated (62). Regarding ATM origins, although initially not included in macrophage fate mapping studies, recently, it was suggested that, under homeostatic conditions, Res-ATMs derive from yolk-sac progenitors (63). However, BM progenitors were also shown to engraft and give rise to Res-ATMs as ~40% of ATMs were derived from the BM following congenic donor BM transplantation (63) moreover whether fetal liver monocytes can contribute to the Res-ATM pool remains to be investigated. This contribution of BM monocytes to the total homeostatic Res-ATM pool in lean mice has also recently been confirmed by fate mapping of ATMs using *Ms4a3*-tagging (61). However, how similar the BM derived Res-ATMs are compared with the embryonic-derived Res-ATMs remains to be investigated. Notably, upon discriminating between VAMs, pre-VAMs and DP adipose tissue macs, Silva et al. identified VAMs to be primarily embryonically derived with only minimal input from BM monocytes while pre-VAMs and DP macrophages were largely derived from the BM (62), suggesting that the turnover of the VAMs is relatively slow with between 1 and 10% of the VAMs being monocyte-derived 60 days after shielded irradiation and congenic donor BM transplant when donor BM input in blood monocytes is normalized to 100% (62). Given the relationship between TIM4 expression and residency time of Res-KCs in the liver (11), as VAM2s had lower levels of chimerism than VAM1s and also expressed TIM4 it is tempting to speculate that VAM2s may be the Res-ATMs that have been in the tissue the longest, while TIM4^lo^ VAM1s may be younger recruits (62). Notably, Res-ATMs were also found to proliferate locally in lean mice (63). In addition to local proliferation, Res-ATM numbers are also maintained through the action of cytotoxic type 1 ILCs in adipose tissue which control ATM numbers (64).

In obese adipose tissue, adipocytes enlarge and subsequently die. In response, the number of ATMs is dramatically increased from ~10% of all immune cells in lean mice to ~50% (65, 66), of which, many are found localized with dead adipocytes forming crown-like structures (CLS) in both humans and mice, allowing the macrophages to surround and engulf the adipocytes (67–69). The increase in ATMs derives partially from the recruitment of new macs in a CCL2 dependent manner, and as in NAFLD, it was these recruited macs that were thought to drive local inflammation and insulin resistance (70–72). In addition, local proliferation of ATMs, specifically those located in the crown-like structures (CLS) has also been reported to contribute to the increase in ATM number in obesity (73–75). Until recently the exact nature of these ATMs in obese adipose tissue was unclear. It had been established that obesity led to a switch in macrophage phenotype from a more M2-like phenotype to a M1-like phenotype (76), with the caveats of using this nomenclature *in vivo* withstanding (Figure 1). However, it was unclear if this represented the recruitment of a distinct population of Temp-ATMs or infRes-ATMs with an M1-like phenotype in obesity or if this also represented plasticity of Res-ATMs. In addition, M1-like cells were typically associated with increased inflammation and a negative outcome however, it is also possible that the recruited ATMs in obese adipose tissue may be beneficial for the tissue, adopting a profile that enables the clearance of dying adipocytes (7). Recently, many groups have begun to address these questions using scRNA-seq and multi-parameter flow cytometry approaches (61, 62, 77–80). Hill and colleagues identified 2 subsets of F4/80^+^CD64^+^ ATMs distinguished on the basis of CD9 expression as well as a population of Ly6C^+^CD11b^+^ monocyte-like cells (78). While the monocyte-like cells were increased in frequency in obese compared with lean adipose tissue, CD9^+^ ATMs were restricted to the adipose tissue of the mice fed a HFD and were also identified in obese patient adipose tissue (78). Notably, some of the Ly6C monocytes also expressed CD9 suggesting these could be the precursors for the CD9^+^ ATMs (78). Both populations were shown to derive from the BM using a chimera model, however the origins of the CD9^−^ ATMs were not assessed (78). The authors found the CD9^+^ ATMs to be located in the CLSs, where they had an increased lipid content and a transcriptional profile enriched for proinflammatory and metabolic genes when compared with the Ly6C monocytes. However, how this compared with the CD9^−^ ATMs was not investigated. Fitting with the more complex *in vivo* environment, the CD9^+^ ATM transcriptional profile was clearly distinct from M1 or M2 profiles (78).

**Figure 4:**
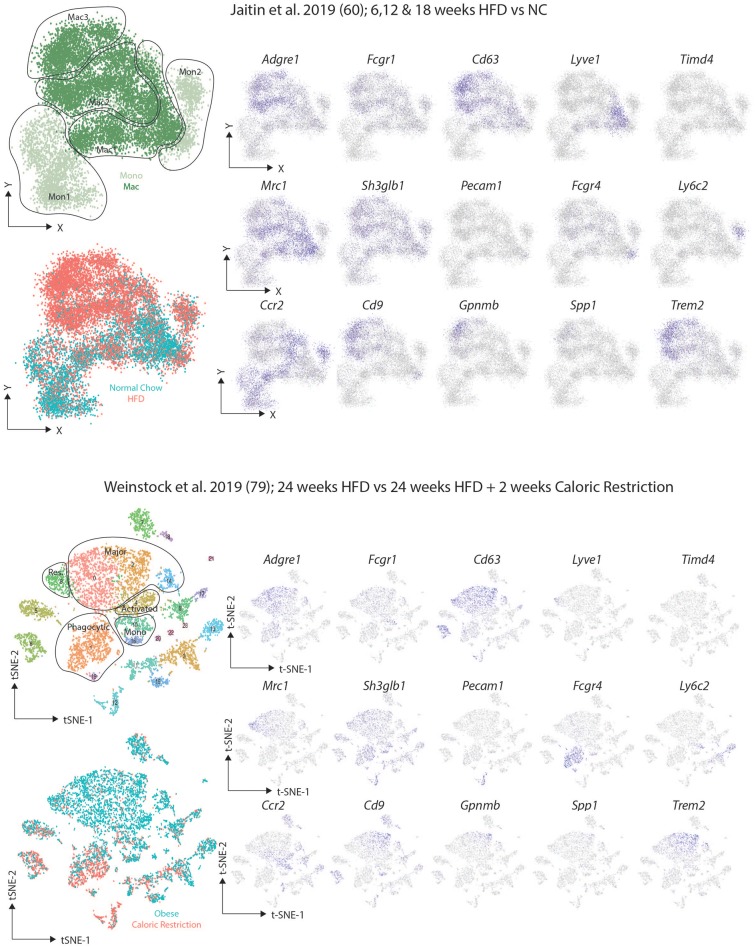
Comparison of murine scRNAseq datasets on macrophages in obese adipose tissue. Publicly available scRNA-seq datasets from (61, 80) were downloaded and tSNE maps recreated as close to the original as possible with the information provided. The distinct clusters of macrophages and KCs as identified in the original studies (61, 80) were identified and expression of the indicated markers assessed.

After identifying the VAM, pre-VAM, and DP mac populations in lean mice using flow cytometry, Silva and colleagues then put the mice on a HFD for 20 weeks. This led to an increase in all ATM populations identified with the largest increase observed in the DP macs (62). Interestingly, these DP macs were reported to correlate with the CD9^+^ macs identified by Hill and colleagues (62), however, Silva and colleagues also reported increased CD9 expression in the VAMs upon HFD feeding suggesting that the CD9^+^ population identified by Hill may include these two subsets (62). In another study, Jaitin et al. identified 3 CD63^+^ ATM clusters using scRNA-seq (61). One cluster, termed Mac1, were the only ATM population present in lean mice and hence were identified as the Res-ATMs (61). This Mac1 cluster contained *Cd9*^−^ ATMs that expressed genes previously associated with perivascular macrophages in the lung interstitium including *Cd163, Lyve1*, and *Retnla* (61, 81), and thus likely correspond to the steady state VAMs (62). Indeed upon downloading the data and regenerating the plots to compare the studies (Figure 4), expression of the proposed VAM markers *Mrc1* (encoding CD206), *Shglb3* and *Timd4* (62) was observed in this *Lyve1*-expressing Mac1 population, although *Shglb3* expression was not restricted to these cells (Figure 4). Notably no second cluster of DP macs was identified in lean conditions again questioning if these truly represent a distinct subset of macrophages or rather a distinct developmental stage/monocyte intermediate given that BM monocytes were found to engraft (albeit slowly) into the VAMs (61, 62). The other two clusters (Mac2 and Mac3) identified by Jaitin et al. expressed *Cd9* and were only found in obese mice, either due to feeding a HFD or genetic defects (db/db obese mice) (61). The Mac2 cluster also expressed the genes encoding surface markers proposed for VAMs (Figure 4) and thus could represent the CD9^+^ VAMs reported to be present in obese adipose tissue (62). However, the Mac2 cluster lacked expression of the genes associated with being located in a vascular niche and rather CD9^+^ macrophages (Mac2 and Mac3) were found to be located in the CLSs (61). If the Mac2 cluster do represent CD9^+^ VAMs, they could be seen as either activated Res-ATMs or infRes-ATMs. As fate-mapping studies using the *Ms4a3*-CrexTdTomato reporter mice found an increased contribution of monocytes to the total CD63^+^ ATM pool after 16 weeks of HFD feeding compared with mice fed normal chow for the same period (61), this could suggest that these would be infRes-ATMs, however, this needs to be tested. Interestingly, the Mac3 cluster, while expressing some genes encoding VAM markers, also expressed *Itgax* (encoding CD11c) and *Mrc1* expression (encoding CD206) was lower than in other Mac populations which could suggest that these are the DP macs identified by Silva and colleagues but then restricted to the obese adipose tissue in this study (61, 62). Overall, while there is potential to align these populations, it is clear that further analysis combining protein and mRNA expression at the single cell level will be necessary to completely align the subsets/clusters identified in these studies and to determine which represent infRes-Macs and Temp-macs in the obese tissue. Further analysis of the differences between the 3 ATM clusters in the study by Jaitin et al. identified the Mac3 cluster to be enriched for a number of genes associated with lipid metabolism and phagocytosis, including *Trem2, Gpnmb, Lipa, Lpl, Cd36*, and *Fabp4* and thus these cells were termed lipid-associated macrophages (LAMs) (61). Although some *Trem2* expression was also observed in the Mac2 population (61). Moreover, the LAMs also expressed the chemokine osteopontin (encoded by *Spp1*) and crucially were also found in human obese adipose tissue (61).

Simultaneously, two additional studies performed scRNAseq studies in obese adipose tissue. These studies identified multiple clusters of macrophages in obese mice including those they termed major macs, activated macs, resident macs and phagocytic macs (79, 80). Major macs were the subset that were most significantly expanded in obese conditions (79, 80) while phagocytic macs were only found in obese adipose tissue and were further expanded upon caloric restriction in obese mice (80). Intriguingly, a cluster within the major macs were enriched for expression of *Cd9, Trem2, Gpnmb*, and *Spp1* (80) (Figure 4), markers of the LAMs identified by Jaitin et al. (61). As lean controls from the same facility were not included in this study it will be interesting to examine if this cluster of major macs are indeed restricted to obese conditions. Similarly, it will be interesting to determine the longevity of this cluster and expression of these genes in more long-term caloric restriction studies as some macs with this LAM phenotype were also found in this study after 2 weeks of caloric restriction (Figure 4) (80).

The presence of phagocytic macs in obese and caloric restricted mice but not lean mice is also interesting (80). A comparison between these and the macrophage subsets identified by Silva and colleagues (62), found that these most closely resembled the DP macs (80), a population of cells difficult to accurately identify in the dataset from Jaitin et al. (61). The two markers proposed to identify the phagocytic macs based on mRNA expression but also at the protein level were *Pecam1* (encoding CD31) and *Fcgr4* (80). While *Pecam1* expression was low in both datasets (Figure 4), *Fcgr4* was indeed enriched in the phagocytic macs from the Weinstock study and was also expressed by the Mac2 cluster in the study from Jaitin et al. (Figure 4). As this cluster is only found in obese adipose tissue fitting with the description from Weinstock et al. perhaps these represent the phagocytic macrophages however, this Mac2 subset does not express *Itgax* (encoding CD11c) suggesting they are likely not the DP macs as described above (at least based on mRNA expression), and thus further comparison of these datasets is warranted. As the phagocytic macs are expanded in caloric restriction, it is also possible that this subset does not exist in the dataset from Jaitin et al., but then the suggestion that the phagocytic macs could be the DP macs should be further examined (61, 62, 80). Notably, some monocytes in the study by Jaitin et al. also expressed *Fcgr4* further questioning if the DP macs in lean adipose tissue represent a distinct subset or rather a developmental intermediate (Figure 4).

**Figure 5:**
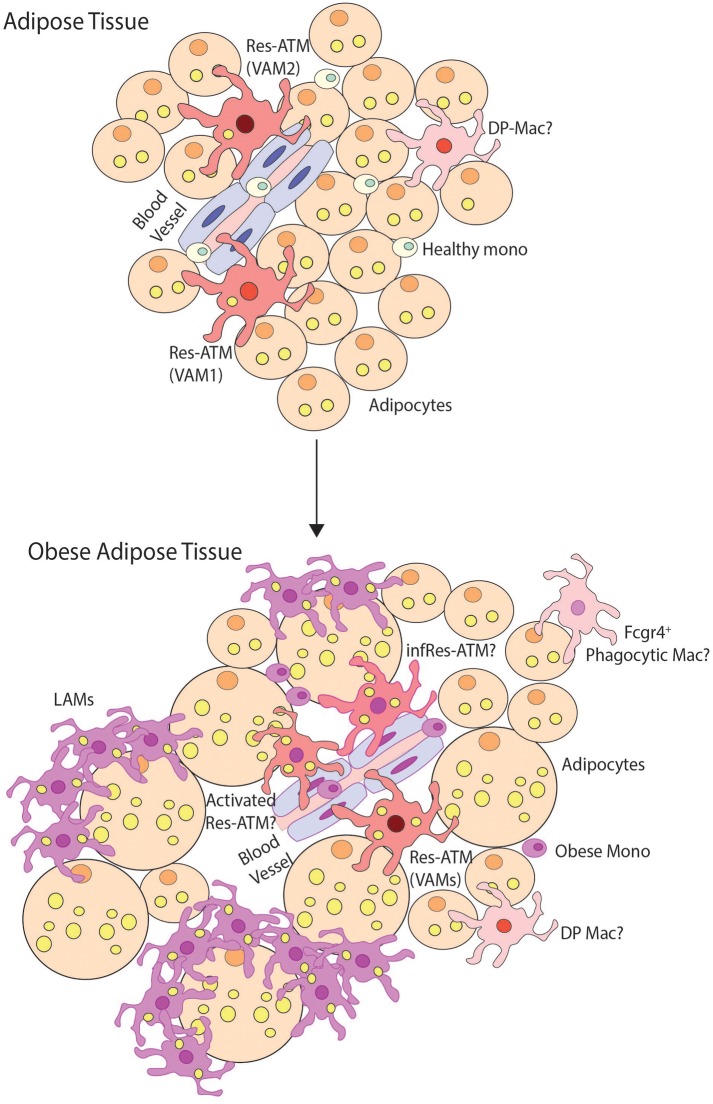
Macrophage heterogeneity in obese adipose tissue. This figure demonstrates possible macrophage heterogeneity present in healthy and obese adipose tissue. In healthy tissue, resident macrophages (Res-ATMs) have been described which express genes suggesting they would be found close to vasculature (61, 62, 80). In one study these were hence termed vascular associated macrophages (VAMs) and could be identified in two subsets those expressing TIM4 and intermediate levels of MHCII (VAM2) and those with a TIM4^lo^MHCII^hi^ profile (VAM1) (62). In addition a population of macrophages deriving from monocytes and expressing CD11c and CD64 termed double-positive macrophages (DP-macs) were also described, however their precise nature remains to be seen as a distinct subset of such macrophages was not observed in healthy adipose tissue in scRNA-seq studies (61, 80). Monocytes are also found in healthy adipose tissue. In obese adipose tissue, the number of monocytes and macrophages dramatically increases. In terms of subsets, Res-ATMs remain, however, exactly how distinct these are from those in healthy tissue remains a question. Some changes in transcriptional profile were identified in VAM1s and VAM2s but whether these represent activated Res-ATMs or infRes-ATMs remains to be seen. Whether some Res-ATMs exist that do not react to the obese environment also remains a question but notably in both scRNA-seq studies, Res-ATMs were identified in the same cluster with those isolated from lean mice (61, 80) suggesting that at least a proportion of the population may not react extensively to the environment. In addition, a population of *Cd9, Trem2 Gpnmb, Spp1* expressing lipid-associated macrophages (LAMs) have also been identified forming crown-like structures in obese adipose tissue. These represent a unique population in the scRNA-seq studies found only in obese adipose tissue. In addition a population of phagocytic macrophages have also been described in obese adipose tissue and being expanded calorie restricted obese mice (80) that have a phenotype similar to DP-macs but also show some overlap with CD9^+^ Temp-macs (non-LAMs) in obese adipose tissue. Thus, the exact nature of these cells also requires further analysis.

While there is still much to learn regarding the macrophage clusters in obese adipose tissue, there is clearly heterogeneity within adipose tissue macs (Figure 5) and a consensus for identifying these populations has not yet been reached. Nevertheless, the LAMs represent a very interesting population. Firstly, they do not appear to be restricted to adipose tissue, as the LAM profile was found to be highly similar to disease-associated microglia (DAMs) found in the brain in Alzheimer's disease (82) suggesting this may be a general of phenotype of macrophages that have to process significant levels of ectopic lipid. Indeed the transcriptional profiles of NAMs (27) and SAMs (28) identified in the NASH or fibrotic liver also bear some resemblance to the LAMs, although exactly how similar these are remains to be studied. Moreover, a population of macrophages in CLSs with a similar transcriptional profile to LAMs including *Spp1, Fabp5, Lpl, Lipa*, and *Cd36* expression was identified during *de novo* lipogenesis induced by β3-adrenergic receptor activation using scRNAseq (77). *De novo* lipogenesis in WAT requires macrophages to clear dead/dying white adipocytes suggesting that this profile could be driven by uptake of adipocytes and dying cells and a need for lipid metabolism. Interestingly, this LAM profile is distinct from Res-KCs despite their expression of genes involved in lipid metabolism (21, 23) and LDAMs, which were identified on the basis of their lipid content and unique transcriptional profiles (26), thus perhaps the signature is governed by more than the presence of lipid. It will be intriguing to understand which other diseases induce such a profile in the recruited macs across tissues. In addition, functionally the LAMs appear to be an interesting population. Given their expression of *Trem2* within the adipose tissue, the role of *Trem2* in the LAMs was also investigated using *Trem2*-deficient mice (61). Notably, LAMs were not identified in the adipose tissue of *Trem2*-deficient mice fed the HFD, with ATMs retaining the signatures of the macs found in clusters 1 and 2, suggesting that TREM2 signaling is specifically required for the LAM profile (61). Notably, the *Trem2*^−/−^ ATMs also had decreased lipid content and the formation of CLSs was abrogated in the absence of *Trem2*-expressing ATMs (61). This correlated with adipocyte hypertrophy and increased weight gain/body fat accumulation and increased signs of metabolic syndrome including glucose intolerance, elevated serum insulin levels and hypercholesterolemia (61). Conversely, deletion of Netrin-1 in macrophages (driven by LysM-Cre) resulted in a partial protection from diet-induced obesity and improved insulin sensitivity coupled with an increase in adipocyte beiging markers (79). This improved outcome correlated with alterations in the macrophage populations including an increase in the proportion of major macrophages compared with the other mac populations (although the number of macrophages in total was reduced compared with WT mice fed the HFD) (79). While an increase in genes associated with lipid handling in the macrophage pool and a decrease in pro-inflammatory lipid mediators were attributed to the improved outcome on the diet (79), as the major mac population appears to include the LAM population (80) and macs of this phenotype have been implicated in *de novo* lipogenesis (77), it is tempting to speculate that this population maybe also plays a role in the improved response to the HFD in the myeloid-specific Netrin-1-deficient mice (79). While this remains to be investigated, it is plausible that, contrary to the original idea that these recruited macs would be detrimental for disease, LAMs may be required to help restrict disease progression. Given that these results were obtained in either full body KOs or chimeras (*Trem2*) or in all myeloid cells (*Netrin-1*) and as macs with similar profiles have been identified in other tissues, it will now be important to examine the role of TREM2 and the expression and function of NETRIN-1 in the distinct macrophage populations in adipose tissue and liver to determine exactly which macrophage populations are important for these differences in disease pathology.

## Concluding Remarks

Taken together, it is clear that there is significant heterogeneity within the macrophage pool in both obese adipose tissue and the fatty liver. This heterogeneity is made up of different subsets (Res-macs, infRes-macs and Temp-macs) but also likely include developmental intermediates and/or different activation states of the same cell subset. While single-cell technologies have helped us to understand this heterogeneity, questions regarding the specific functions of these different subsets and/or the precise definition of distinct subsets remain which will be important to understand in the coming years. Studies using full body KOs and chimeras are helping to shed light on the functions of the different populations, but further work is still required using cell-type specific KOs before we can truly assess the roles of one population over another so that we can better understand which populations we may need to target with new therapeutic approaches.

## Author Contributions

AR and CS co-wrote the review. LM performed the bioinformatics analysis.

## Conflict of Interest

The authors declare that the research was conducted in the absence of any commercial or financial relationships that could be construed as a potential conflict of interest.

## Supplementary Material

Table S1 Summary of scRNA-seq studies.

Table S2 Genes associated with M1/M2 modules and Res-macs.
